# An Unexpected Innocent Complication Associated with Azacitidine Treatment of Myelodysplastic Syndrome: Erythema Annulare Centrifugum

**DOI:** 10.4274/tjh.2015.0268

**Published:** 2016-02-17

**Authors:** Esra Turan Erkek, Sevgi Kalayoğlu Beşışık

**Affiliations:** 1 İstanbul University İstanbul Faculty of Medicine, Department of Internal Medicine, Division of Hematology, İstanbul, Turkey

**Keywords:** Erythema annulare centrifugum, Azacitidine, myelodysplastic syndrome

## TO THE EDITOR

Skin lesions accompanying hematological malignancies can be formed due to either direct tumor infiltration of the skin or indirect effects. Indirectly developing lesions may be a component of paraneoplastic syndrome. Erythema annulare centrifugum (EAC) is considered to be a hypersensitivity reaction developed against various antigens associated with infections, drugs, and endocrine diseases. EAC, rarely seen in neoplastic diseases, has been reported in lymphoma, leukemia, histiocytosis, and prostate cancer. Here we report EAC in a patient using a hypomethylating agent, azacitidine.

**Figure 1:**
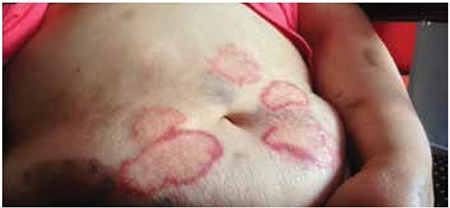
Erythema annulare centrifugum rashes formed during treatment.

**Figure 2:**
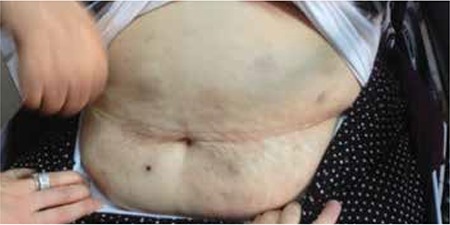
Skin lesions’ regression after treatment interruption.

A 69-year-old female patient was admitted to our polyclinic with weakness and ecchymosis in her legs existing for 3 months. She was considered as having refractory anemia with excess blasts-2 according to myelodysplastic syndrome (MDS) classification [1]. Because there was only hyperdiploidy in conventional cytogenetic examination, she was classified in group intermediate-2 of the International Prognostic Scoring System. She had a history of radical mastectomy and adjuvant chemoradiotherapy for breast cancer 3 years ago. She said that variously sized round and oval erythematous, itching, painless lesions had formed in the abdominal region on the 4th day of azacitidine usage (75 mg/m2/day, 7 days, s.c.) (Figure 1 and 2). There were no concomitant complaints or physical examination findings except fatigue. After azacitidine was stopped, a skin biopsy was taken. In the biopsy, mild perivascular inflammatory infiltration accompanying vascular ectasia in the papillary dermis was detected. The possibility of paraneoplastic syndrome was excluded due to the disappearance of all lesions by 1 week after cessation of treatment. During the second course of azacitidine, the lesions reoccurred on the second day. Subsequently to the second course, the patient died of sepsis, which developed after pneumonia.

EAC was first defined by Darier in 1916, and it was classified into categories of superficial and deep forms by Ackerman in 1978 [2]. In the deep form, the lesions are hard and are usually seen together with desquamation without itching. The superficial form is characterized by itchy lesions with uncertain borders and desquamation. EAC formation is associated with trauma, ectoparasites, tuberculin test, PUVA therapy (photochemotherapy), viral infections, and diabetes. There are publications reporting that EAC may be associated with Hodgkin’s lymphoma rarely and lung, colon, cervix, prostate, stomach, and ovarian cancers even more rarely [3,4]. Lesions are often observed on the trunk, proximal portions of the limbs, and the buttocks. Today EAC is defined as a characteristic hypersensitivity reaction that can be triggered by many different antigens and disappears within 1-2 weeks.

Skin lesions, whose most common forms are Sweet syndrome and myeloid sarcoma, are rarely observed in MDS [5]. Azacitidine, a nucleoside analogue, is one of the low-density treatment options in MDS. Azacitidine usage may cause cutaneous reactions such as urticaria, skin dryness, nodules, localized hematoma at the injection area, rash, granuloma, swelling, pigmentation changes, and induration.

This case was presented because no AEC development during azacitidine use in MDS had been reported previously.
